# 

**Published:** 1896-12

**Authors:** 


					﻿CONTENTS.
ORIGINAL ARTICLES—
Diseases of the Rectum and Anus. By George K. Sims,M. D.,
Richmond, Va............................. 589
A Review of the Author’s Method of Anchoring the Kidneys.
By Harvey Reed, M. D., Columbus, Ohio... 600
SOCIETY REPORTS—
Tri-State Medical Society of Alabama, Georgia and Tennes-
see...................................... 603
SELECTIONS AND ABSTRACTS—
Solvent Properties of Buffalo Lithia Water.... 624
EDITORIAL .................................... 630
SPECIAL NOTES..............................    631
PRESCRIPTIONS................................  641
				

